# Nigellothionins from Black Cumin (*Nigella sativa* L.) Seeds Demonstrate Strong Antifungal and Cytotoxic Activity

**DOI:** 10.3390/antibiotics10020166

**Published:** 2021-02-06

**Authors:** Anna S. Barashkova, Vera S. Sadykova, Victoria A. Salo, Sergey K. Zavriev, Eugene A. Rogozhin

**Affiliations:** 1 Shemyakin and Ovchinnikov Institute of Bioorganic Chemistry Russian Academy of Sciences, 16/10, ul. Miklukho-Maklaya, 117997 Moscow, Russia; szavriev@ibch.ru (S.K.Z.); rea21@list.ru (E.A.R.); 2 Gause Institute of New Antibiotics, 11 ul. Bol’shaya Pirogovskaya, 119021 Moscow, Russia; sadykova_09@mail.ru; 3 Laboratory of Molecular and Cellular Biophysics, Sevastopol State University, 33 Universitetskaya Str., 299053 Sevastopol, Russia; viktoriya_salo@mail.ru; 4 All-Russian Institute of Plant Protection, Pushkin, 196608 St. Petersburg, Russia

**Keywords:** thionins, *Nigella sativa*, plant antimicrobial peptides, amino acid sequence, antifungal activity, cytotoxic properties

## Abstract

High-cationic biologically active peptides of the thionins family were isolated from black cumin (*Nigella sativa* L.) seeds. According to their physicochemical characteristics, they were classified as representatives of the class I thionin subfamily. Novel peptides were called “Nigellothionins”, so-called because of their source plant. Thionins are described as components of plant innate immunity to environmental stress factors. Nine nigellothionins were identified in the plant in different amounts. Complete amino acid sequences were determined for three of them, and a high degree of similarity was detected. Three nigellothionins were examined for antifungal properties against collection strains. The dominant peptide, NsW2, was also examined for activity against clinical isolates of fungi. Cytotoxic activity was determined for NsW2. Nigellothionins activity against all collection strains and clinical isolates varied from absence to a value comparable to amphotericin B, which can be explained by the presence of amino acid substitutions in their sequences. Cytotoxic activity in vitro for NsW2 was detected at sub-micromolar concentrations. This has allowed us to propose an alteration of the molecular mechanism of action at different concentrations. The results obtained suggest that nigellothionins are natural compounds that can be used as antimycotic and anti-proliferative agents.

## 1. Introduction

Plants are subjected to a wide range of abiotic and biotic stresses and have evolved various molecular defense mechanisms. Antimicrobial peptides (AMPs) are a part of plants’ innate immunity [1]. A wide range of plant AMPs have been described and grouped into families: thionins, defensins, hevein-like, knottin-type, lipid transfer proteins, α-hairpinins, snakins, cyclotides, and some unclassified peptides [2]. Most AMPs are cationic, cysteine-rich molecules, stabilized by disulfide bonds. Thionins were the first plant AMPs to be described. Balls et al. first crystallized thionins in 1942 [3] and Okada et al. described them as a “lethal toxic substance for brewing yeast” in 1970 [4]. Thionins are small (~5 kDa) cationic peptides divided into five classes based on the number of cysteine residues and charge. Thionins with eight cysteine residues are divided into classes I and II, and thionins with six cysteine residues into classes III, IV, and V. The sequences and secondary structures of thionins are highly conserved. The secondary structures of thionins generate a “β1-α1-α2-β2-random coil” motif with the N-terminus forming the first β-sheet [5]. The spatial structure of thionins has shown a distinct capital Greek gamma letter “*Γ*” shape. The vertical arm consists of two antiparallel α-helices, and the horizontal arm contains a coil in an extended conformation and a short antiparallel β-sheet. Thionins typically have an amphipathic character with hydrophobic residues located on the outer surface of the two α-helices. Hydrophilic residues are situated on the undersurface of the “*Γ*” and at the outer surface of the corner of the “*Γ*”, as well as on the inner surface of the molecule, where positively charged residues are concentrated [4,6]. Thionins have been found in monocots (*Poaceae* and *Liliaceae*) and dicots (*Santalaceae*, *Ranunculaceae*, *Viscaceae*, *Brassicaceae*) [3,4,7]. Thionins are considered plant toxins because of their toxic properties against a wide range of prokaryotic and eukaryotic organisms, as well as mammalian cells [8,9,10].

Previously, we reported two novel thionins from black cumin (*Nigella sativa* L.) with antimicrobial properties against Gram-positive bacteria and yeasts [11]. Here, we describe the molecular diversity of *N. sativa* thionins and provide data on their antifungal and anti-proliferative properties.

## 2. Results

### 2.1. A New Subfamily of the Antimicrobial Peptides Isolated from N. sativa Seeds Is Represented by Plant Thionins with 8-Cys Motif (Thionin I Class)

**Figure 1:**
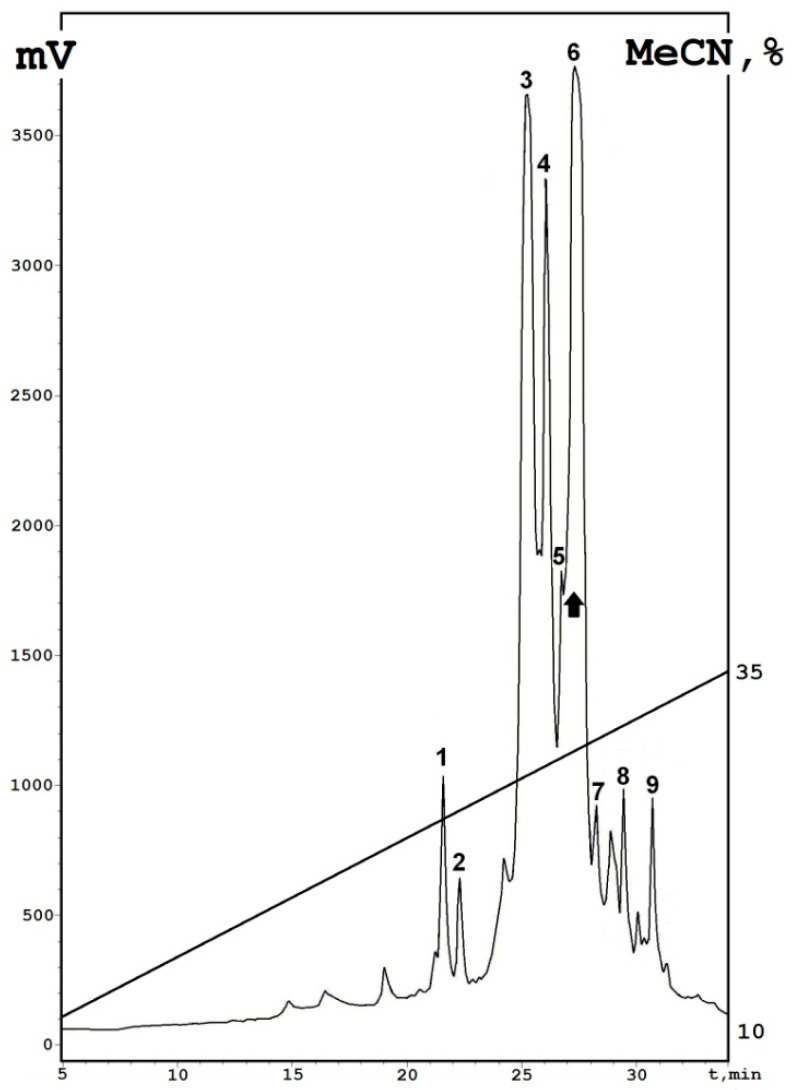
Reversed-phase HPLC (RP-HPLC) profile of 1 M fraction (fraction 4) after affinity chromatography on a Heparin-Sepharose column, washed with buffer containing 1M NaCl. All collected fractions characterized are indicated by ciphers; a dominant peak is marked by a black arrow. Axis: X—time, min; Y—is a digital signal axis (mAU, milli Absorbance Units).

Here, we used an approach similar to that used in our previous work when two antimicrobial thionins (NsW1 and NsW2) were isolated [11]. One significant change was applied. Fraction 4 (where thionins were found) did not contain any high-molecular-weight proteins; this allows us to reduce the size-exclusion chromatography step. The resulting scheme of the thionins isolation from *N. sativa* extract consists of affinity FPLC and reversed-phase HPLC of the most bounded fraction 4 (eluted by 1 M NaCl) (Figure 1). Nine fractions were collected: three major (3, 4, and 6), and six minor (1, 2, 3, 7, 8, and 9).

All compounds obtained were initially characterized and identified by HPLC/ESI-MS and N-terminal sequencing (20 amino acid residues were determined) (Table 1; Figure S1). Fractions were eluted from the HPLC column during the time interval of 25 to 31 min. The molecular masses were measured in a range of 5.0–5.3 kDa. The N-terminal amino acid sequencing coupled with BLASTP searches allowed us to identify all samples as thionins (called NsW1–9) with an 8-Cys motif (class I/II), characteristic of the family of antimicrobial peptides annotated as defense molecules of plant innate immunity. They are constitutive and inducible (PR-proteins class 13) [2,12]. Two out of three major peptides (fractions 3 and 6) were identified as NsW1 and NsW2, reported in our previous paper [11]; another peptide (fraction 4) is a novel peptide termed NsW4. A single amino acid substitution Met19Ile at position 19 was found in NsW2–4. A unique Lys1Gly substitution was found in NsW8–9. NsW8–9 differ from other homologues isolated from all plant thionins reported previously [13]. Thus, black cumin (*N. sativa*) seeds contain a group of highly homologous peptides belonging to the thionin family with an 8-cysteine motif; most of these are expressed in low quantities. Based on their partial sequences, we hypothesize that these peptides compose a subfamily.

Three major compounds (NsW1, NsW2 and NsW4) were collected for further experiments. It was shown that there is no contamination, and peptides are chromatographicaly pure (Figure S2.). The total yield of these peptides per 1 g of seeds was 15 µg NsW2, 11 µg NsW1 and 2.7 µg NsW4.

**Figure 2:**
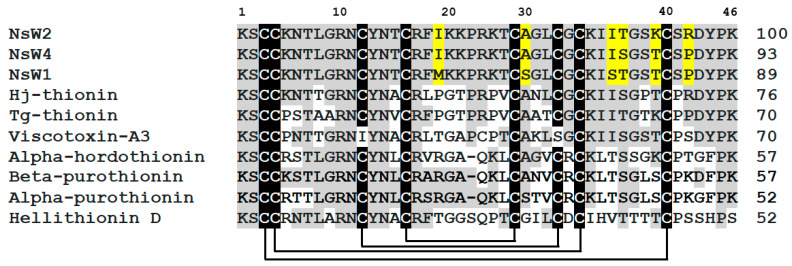
Multiple sequence alignment of nigellothionins and most homologous plant peptides deduced based on a BLASTP algorithm: NsW1, NsW2, and NsW4 from *N. sativa*; Hj-thionin from *Hordeum jubatum* [14]; Tg-thionin from *Tulipa gesneriana* (GenBank ID: CAA57353.1); viscotoxin A3 from *Viscum album* [15]; alpha-hordothionin from *Hordeum vulgare* [16]; Alpha-purothionin from *Triticum aestivum* [17]; hellithionin D from *Helleborus purpurascens* [18]. Cysteine residues are indicated by a white type on a black background; all identical residues are colored white-gray (relative to the NsW2 structure, which is outlined with a frame); amino acid substitutions between the thionins from *N. sativa* are marked in yellow. The degree of sequence identity (relative to the NsW2 structure) is represented by ciphers.

The complete sequences of the predominant thionins (NsW1, NsW2, and NsW4) were determined by Edman automated sequencing. All these peptides consist of 46 amino acid residues, including eight cysteine residues that form four disulfide bridges. They are identical and have local structural differences represented by single amino acid substitutions (Ile19Met, Ala27Ser, Ile35Ser, Trn36Ser, Lys39Trn, and Arg42Pro) (Figure 2). There is high sequence homology among them (89–93% relative to NsW2 sequence). Searching the sequence homology of NsW1, NsW2, and NsW4 to other thionins annotated in UniProtKB/SwissProt databases showed the differences in sequence similarity (Figure 2). We found that the nigellothionins have relatively low homology (from 52 to 76%) to thionins from other plants.

Surprisingly, hellithionin D isolated from purple-red (*Helleborus purpurescens*) [18] was not similar to *N. sativa* thionins even though both plants belong to the *Ranunculaceae* family. At the same time, viscotoxin-A3, a member of the 6-cysteine thionins subfamily, demonstrates 70% homology with nigellothionins, which is very unusual. Concerning local differences in the amino acid sequences, a site between Cys4 and Cys5 represented by two α-helices connected with β-turn shows the maximal structure variability in plant thionins. For instance, α-purothionin [19] has a maximal variable amino acid substitutions in relation to nigellothionins and all other homologous peptides. One more differential peculiarity is associated with Pro42Arg substitution in the NsW2 structure relative to other nigellothionins and thionins from other plants. The C-terminus for all plant thionins is represented by a random coil that is consequently unstructured. However, this fragment contains two proline residues providing a spatial turn. The above-mentioned substitution can affect the three-dimensional orientation of the NsW2 peptide. These traits might be critical with respect to further structure-functional investigations in terms of modes of action of nigellothionins. Finally, we note that nigellothionins are much less similar to all homologous species found and phylogenetically stand out on a separate branch (Figure S3).

### 2.2. Thionins from N. sativa Seeds Demonstrate Growth Inhibitory Activity against Filamentous Fungi and Yeasts

Three nigellothionins (NsW1, NsW2, and NsW4) isolated from *N. sativa* seeds were tested against conditionally pathogenic *Aspergillus* strains to reveal their antifungal activity (Table 2). All thionins tested have a comparative antifungal activity towards three of four fungal species (*Aspergillus flavus* 3K, *A. fumigatus* 2K, and *A. oryzae* 1K).

**Figure 3:**
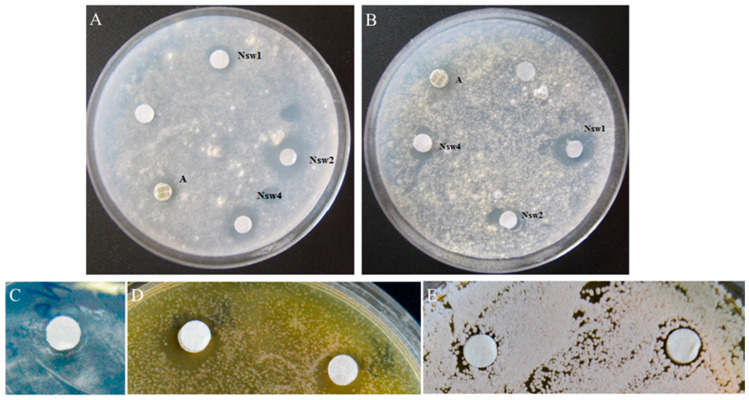
Antimicrobial activity of the *N. sativa* thionins (NsW1, NsW2 and NsW4) detected by disc-diffusion method: *A. fumigatus* 2K (**A**) and *A. flavus* 3K (**B**); Amphotericin B was used as positive control. Inhibition caused by NsW2: *A. fumigatus* 163m (**C**), *Cryptococcus laurentii* 801M (**D**) and *Candida krusei* 1447N (**E**); Nystatin and Fluconazole were used as positive control (not shown on photo).

Antifungal activity of NsW2 and NsW4 against *Aspergillus* spp. was equal, whereas NsW2 was less active towards *A. fumigatus* 2K and *A. oryzae* 1K but not on *A. flavus* 3K. The activity of all peptides tested against *A. flavus* was comparable to reference polyene cyclic antibiotic AmB (Figure 3). Interestingly, none of the major nigellothionins inhibited the growth of *A. terreus* or of the control antifungal drugs.

Nigellothionin NsW2 was selected for further functional analysis because its amount was sufficient. The selected nigellothionin was tested against several clinical isolates of *Aspergillus* and *Candida*, which are causative agents of invasive aspergillosis and candidiasis of patients with pulmonary tuberculosis. The disc-diffusion method at the active concentration of 19.4 µM (~1 nmol/per disc; Figure 3) was used.

NsW2 was active against all clinical fungal cultures tested. The level of growth inhibition by NsW2 against most fungi was approximately equal between the variants and comparable to the cultures from the collection (Table 3). It is important to note that nigellothionin NsW2 was active towards the clinical isolate of *A. terreus* while it was inactive on the culture from the collection. *A. terreus* has an exceptional position within the genus *Aspergillus* due to its intrinsic resistance against the polyene AmB. Our results confirm the complete resistance to AmB for both of the *A. terreus* cultures previously tested [20].

We determined MIC values for NsW2 toward collection and clinical *A. fumigatus* cultures using a microtiter plate assay to understand the antibiotic potential of this natural plant peptide. The MIC value was 0.77 µM for both isolates.

NsW2 was tested against triazole-resistant yeasts with decreased susceptibility to statins. Thionin showed moderate activity against *Sacharomyces. cerevisiae* 77S, *Cryptococcus laurentii* 801M, and *Candida krusei* 1447N (Table 4). It is worth noting that *C. glabrata* 1402m was not sensitive to this compound. All other cultures tested were suppressed by the addition of the NsW2 peptide. The inhibition zones were similar to AmB.

Invasive pulmonary aspergillosis treatment is initiated after conidia germination in patients; thus, it is important to identify a compound with fungicidal activity against the hyphal elongation of germinated conidia of *Aspergillus* species. We monitored growth dynamics for three clinical isolates (*A. terreus* 1133, *A. fumigatus* 163m, and *A. flavus* 905m) under the nigellothionin time-dependent decreasing of activity after 1, 2, and 5 days of incubation. The effectiveness of the NsW2 decreased over time. All fungal cultures tested were inhibited by thionin after 1 day of incubation.

The dynamics of the activity decline were similar for all *Aspergillus* isolates tested. Upon treatment, hyphal elongation increased in proportion to the incubation time. The area of mold increased in proportion to hyphal elongation. Nigellothionin lost activity on the second day of the incubation, unlike the AmpB.

### 2.3. Cytotoxic Activity Caused by the NsW2 Thionin from N. sativa towards Tumor and Normal Cell Lines In Vitro

Cells from lines B16 (mouse melanoma), HTC-116 (human adenocarcinoma), and human postnatal fibroblasts (HPFs) were treated by the peptide.

Cells were cultivated at a range of NsW2 concentrations. The nigellothionin affected all cell lines. According to the IC50 values (Table 5), we conclude that there was no significant difference between the activity of NsW2 on various tumor cell lines. In contrast, the cytotoxic activity on the normal HPF cells was lower.

We observed a different pattern of cellular response to the NsW2 peptide incubation. HCT-116 cells showed an obvious concentration-dependent reaction on NsW2; the viability of HPF and B16 cells decreased by up to 50% and remained at the same level regardless of the increase in NsW2 concentration. The cell lines’ sensitivities to doxorubicin were not different. Thus, there is a relatively direct proportion between NsW2 and doxorubicin action towards HCT-116 cells, whereas the reactions of HTC-116 and HPF on doxorubicin were similar. Cell viability after NsW2 treatment decreased in a concentration-dependent manner. B16 cells were resistant to this compound up to 0.8 µM (Figure 3); their viability then fell to the same level as other cell lines.

**Figure 4:**
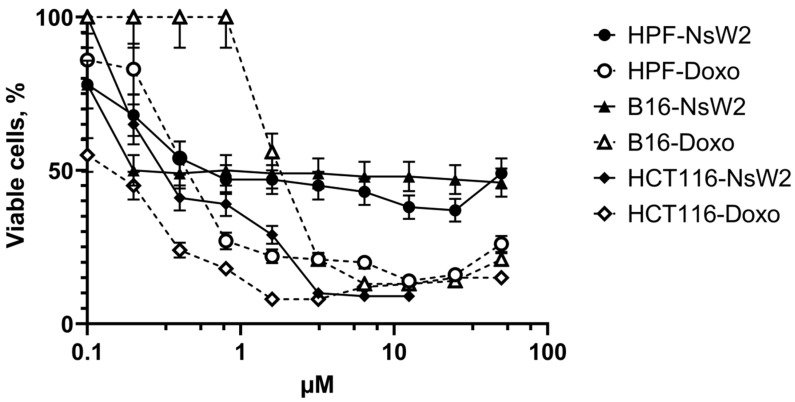
Cytotoxic activity of NsW2 in vitro. Doxorubicin was used as a positive control. Concentration dependence is shown by a solid line with black dots; the activity of doxorubicin is shown by a dashed line with white dots (♦) HPF cell line; (◊) HPF cell line with doxorubicin; (■) B16 cell line with NsW2; (□) B16 cell line with doxorubicin; (●) HTC-116 cell line with NsW2; (○) HTC-116 cell line with doxorubicin. Error bars represent mean ±S.E. (three independent experiments performed in triplicate).

To identify possible relationships and the specific mode of NsW2 action on eukaryotic cell lines, we also tested this thionin on human buccal epithelium cells to evaluate heterochromatin condensation as the indicator of replication activity inside the cells. The reliable effect of NsW2 was detected at 0.4 µM (Figure S4). This corresponds to IC_50_ values with data estimated experimentally in MTT assays (Figure 4).

## 3. Discussion

Here, we report a diversity of high cationic biologically active thionin peptides localized in black cumin (*N. sativa*) seeds. We also characterize the structure of these active thionin peptides and their biological activities towards eukaryotic organisms (fungi and yeasts) and cell lines in vitro.

Plant thionins possess various functional effects, including antimicrobial and antitumor; the primary mechanism of action is associated with membrane activity [21]. Previously, we isolated and identified two major thionins (NsW1 and NsW2) from black cumin (*N. sativa*). These molecules were found to have antibacterial activity against both Gram-positive and Gram-negative bacteria. We also found plasma membrane permeability and deformation of the cellular surface on yeast [11]. The extraction procedure included water-acidic treatment followed by acetone precipitation of proteins and peptides. After desalting, the extract was fractionated by three-step liquid chromatography (affinity, size-exclusion, and reversed-phase HPLC), and thionins were isolated [11]. It was found that fraction four from affinity chromatography was poor in high-positively charged proteins, so the fractionation procedure was simplified by the reduction in gel filtration. *N. sativa* seeds are rich in basic high-molecular-weight proteins, and some of them are storage proteins [22]. Previously, we reported antimicrobial peptides NsLTP1, belonging to the lipid-transfer proteins, and NsD1/D2, the representatives of plant defensin families. These peptides have been isolated from a total fraction eluted by 100 mM NaCl from heparin-sepharose column (affinity medium-pressure chromatography) [23,24]. NsLTP1 was isolated using a two-step chromatography approach without molecular weight separation. As a result, its quantity and purity were low [25]. NsD1/D2 was not visualized at all. Thus, cation-exchange low-pressure separation onaCM52 column was used to optimize a scheme and remove all storage proteins identified as 7S and 11S globulins according to N-terminal sequencing results (our unpublished data).

As indicated above, in this work, we first isolated and structurally confirmed several homologous thionins with an 8-cysteine motif isolated from a single biological source. Prior to this, viscotoxins as 6-cysteine thionins had been allocated to a separate subfamily [26]. The content of individual thionins was different; based on this point, they were initially classified as “major” (NsW1, NsW2, and NsW4) and “minor” (NsW3 and NsW5–9). Given that thionins are involved in PR-proteins class 13 [12,19], genes encoding them might be inducibly expressed and up/down-regulated depending on the type of stress factor recognized by plant innate immunity [27,28].

Sequence analysis of the dominant nigellothionins (NsW1, NsW2, and NsW4) identified single amino acid substitutions mostly inside the C-terminal fragment, and in the second α-helix. It is unusual that a substitution, localized in position 19 of the polypeptide chain and inserted into a β-hairpin connecting two α-helices, is critical for biological activity—the hydrophobic Met and Ile residues in the NsWs are absent for all other plant thionins containing preliminary positive charged or neutral amino acids. This site is the most divergent, as described earlier. In addition, all of the sequenced nigellothionins have a high positive charge (+9.0 … +11.0) at neutral pH value; thus, they could be classified as the members of thionin class I subfamily, and their functions might be similar to purothionins from cereals [5]. However, it is important to note that an α-helical cysteine motif that is typical for the other group of plant defense peptides (called α-hairpinins or hairpin-like peptides) [29] is integrated into the thionin spatial structure [8]. Consequently, the surface topography for thionins might be similar to the α-hairpinins that determine biological activity: thus, the β-hairpin site inside the structure of plant hairpin-like peptides is a key factor of target biological activities, including antibacterial [30,31], antifungal [32], or trypsin inhibition [33].

The antifungal activity of thionins is well-known. Most studies are devoted to thionin interactions with phytopathogenic fungi. However, there are few studies on their activity against opportunistic fungi and representatives of genus *Aspergillus* causative agents of invasive mycosis and allergy. Nigellothionins NsW1, NsW2, and NsW4 were tested against four *Aspergillus* species. All nigellothionins were active against three of four strains tested, and the activity levels were similar for all peptides. The minor differences of their activity observed might be related to local substitutions in the peptides’ sequences, as indicated above. There was only one study of thionin activity against a representative of the genus *Aspergillus*. Molina et al. [34] tested wheat (*Triticum aestivum*) type I thionins on a wide range of fungi, including *A. nidulans*; the fungus appeared to be sensitive to a mixture of thionins.

Plant thionins are active against phytopathogenic fungi. Thus, thionins isolated from barley leaves (*Hordeum vulgare*) have strong inhibition activity against *Thievaliopsis paradoxa* and *Drechslera teres* [35]. Another 8-Cys thionin isolated from parasitic plant *Pyrilaria pubera* demonstrated activity against *Plectosphaerella cucumerina*, *Fusarium oxysporum f.* sp. *conglutinan*, and *Botrytis cinerea* [8].

The total yield of nigellothionin NsW2 was the highest, and further experiments were performed using this peptide. NsW2 was tested against some clinical isolates of filamentous fungi from the genus *Aspergillus* and yeasts. The activity level for most of the clinical strains tested was similar, as well as the activity against isolates from the collection. There was no significant difference between clinical isolates and all non-clinical strains. In contrast, NsW2 was active against clinical *A. terreus* 1133, whereas wild strain *A. terreus* 3K showed total resistance to the peptides’ action. *A. terreus* 3K was sensitive to AmB, which is consistent with data on high *A. terreus* polyene resistance. The mode of action of AmB is associated with specific interactions with sterols, particularly ergosterol. The subsequent effects of AmpB include membrane permeabilization and induction of oxidative stress [20]. The difference between activity of NsW2 toward wild and clinical strains could be explained by the differences in fungal antioxidant defense system. Thionins induce intracellular reactive oxygen species production (H_2_O_2_), which could be toxic for cells [36]. This is one of the mechanisms of thionin toxicity against fungi [5,21,37]. In the case of NsW2 sensitivity, the fungal antioxidant system is most likely not capable of scavenging all of the free radicals, which subsequently leads to cell death. In the case of NsW2 resistance, the antioxidant system overcomes thionin-induced oxidative stress. However, the absence of NsW2 activity on non-clinical isolates suggests the presence of an alternative mechanism of nigellothionin action on fungal cells.

Thionins can rapidly permeabilize fungal membranes, as indicated in fluorescent dye experiments [38]. Previous studies have reported antifungal activity against *C. albicans* for the nigellothionins NsW1 and NsW2 [11]. Here, four pathogenic yeast isolates were subjected to NsW2 treatment, and three of them were sensitive to peptide action. The anti-yeast activity of thionins is established. *S. cerevisiae* is also used in functional studies as a model organism [38]. In contrast, so-called plant γ-thionins, which have been classified into the defensin family, have anti-yeast activity [39]. This study is the first report of thionin activity against clinical yeast isolates.

Concerning the anti-proliferative activity of the thionins, it should be noted that the cytotoxic effects of 6-Cys thionin viscotoxin from *Viscum album* are well-known. Still, the properties of 8-Cys thionins are relatively less studied despite the proposed anti-cancer drug prototypes that represent one more novel aspect of this investigation. We examined NsW2 for cytotoxic properties on two tumor lines and one non-tumor cell line. NsW2 showed an activity level comparable with purothionins and phoratoxins, according to IC_50_ [10,40,41], but there was no significant difference between the cell lines tested. Most biological activities of thionins are directly related to their interaction with plasma membranes. However, there are also some secondary effects, including interactions with nucleic acids, that are certainly not associated with cell membrane disruption.

It was shown that the critical dose directly associated with toxicity and membrane lysis is 1 μM [5]. Here, we report a significant decrease in cellular viability at a thionin concentration below 1 µM (0.2–0.55 µM). The cytotoxic effects of β-purothionin from wheat (*T. aestivum*) can form ion channels in artificial membranes at 1 µM [42]. These results correlate with those previously determined for other tumor cell lines (Jukart, RD, AsPC-1, and Colo357) that were inhibited at IC50 from 0.36 to 0.75 μM [43]. We suggest an alternative mechanism of thionin toxicity, which takes place at their low concentrations in contrast to the principal membrane-associated mechanism. Here, we confirm this by the heterochromatin condensation in buccal epithelial cells in response to NsW2 treatment at concentrations less than 1 µM. These data indicate that, at low concentrations, NsW2 enters the cellular nucleus and forms complexes with nucleoproteids via electrostatic and hydrophobic interactions. Supportive evidence was obtained in a previous study on direct suppression of some oncogenes in rhabdomyosarcoma cells encoding matrix metalloproteinases (*MMP-7, MMP-13*), miRNA (*Mir21*), transforming protein RhoA (*RhoA*), and apoptosis inhibitor-1 (*IAP-1*)—possible regulators of transcriptional activity in eukaryotic cells [43]. This thesis is also confirmed by the DNA-binding properties of 6-Cys thionins [44,45].

Nigellothionins also possess antifungal effects, but they disappear on the second day of incubation. This can be explained by fermentative hydrolysis of peptides by fungal proteases [46]. EcAMP1, a plant antimicrobial peptide with α-helical structural motif, was previously shown to penetrate fungal conidia [47]. Thus, we can speculate about peptide redistribution among fungal cells and the surrounding media. When NsW2 partially enters the cells, it inhibits fungal growth in a high-concentration manner. The extracellular quantity of this peptide decreases, and the peptide might still act in a “low concentration” manner. Still, the activity of the peptide could not be detected via the disc-diffusion assay.

## 4. Materials and Methods

### 4.1. Microorganisms

#### 4.1.1. Non-Clinical Strains

A collection of the conditionally pathogenic mold fungi of *Aspergillus* genus (i.e., *A. flavus* 3K, *A. terreus* 3K, *A. fumigatus* 2K, and *A. oryzae* 1K) was obtained from the Official Culture Collection of Department of Mycology and Algology, Biological Faculty, Lomonosov Moscow State University (Russia).

#### 4.1.2. Clinical Isolates

The clinical isolates of yeast (*Candida glabrata* 1402m, *C. krusei* 1447m, *Cryptococcus laurentii* 801m, *S. cerevisiae* 77 S) and *Aspergillus* spp. (*A. tereus* 1133 m, *A. flavus* 905 m, *A. fumigatus* 163) from immunocompromised patients with invasive pulmonary aspergillosis and acute invasive candidiasis were taken from the Collection of Mycological Laboratory of the Moscow Municipal Scientific Practical Center of Tuberculosis Control (Russia). All clinical isolates demonstrated azole resistance in vitro to commercial fluconazole and itraconazole.

### 4.2. Isolation of the Thionins from Black Cumin (Nigella sativa) and Structural Studies

#### 4.2.1. Thionins Isolation Procedure

Isolation of the thionins from *N. sativa* seeds was conducted as described previously [11] with minor modifications. After the crushing of the seeds, followed by acetic extraction and precipitation with ice-cold acetone, a total pellet was air-dried and redissolved in excess of 0.1% trifluoroacetic acid (TFA). Then, initial separation by solid-phase extraction was carried out using reversed-phase HPLC on an Aquapore C8 column (10 × 100 mm^2^) (Applied Biosystems, Waltham, MA, USA). The mobile phases were 0.1% TFA (Solvent A) and 80% acetonitrile in 0.1% TFA (Solvent B). After washing of all unbounded organic salts and other hydrophilic components, proteins and peptides were eluted with 75% solvent B. The extract was evaporated using a Speedvac concentrator (Thermofisher, Waltham, MA, USA) and lyophilized. A protein–peptide extract was then redissolved in 0.1 M Tris-HCl (pH 7.2) and applied on a Heparin-HiTrap Sepharose 5 mL (GE HealthCare, Chicago, IL, USA) affinity column for further fractionating by the charge in a stepwise gradient of NaCl. Four combined fractions were obtained: unbound compounds 0 mM NaCl (fraction 1); 100mM NaCl (fraction 2); 500 mM NaCl (fraction 3); 1000 mM NaCl (fraction 4). Fraction 4 (thionin-rich fraction, 1 M NaCl) was separated by analytical reversed-phase HPLC on an XBridge C_18_ (4.6 × 250 mm^2^, 5 µm, 300 Å) column (Waters, Milford, MA, USA) in a linear Solvent B gradient (10–50% B) for 60 min at a flow rate of 0.95 mL/min and 42 °C. The elution of peptides was monitored at 214 nm [11].

#### 4.2.2. Determination of Peptides Purity

Purity of collected peptides was checked by RP-HPLC on an analytical column Luna C_18_ 100 × 2.0, 3 µm, 100Å (Phenomenex, Torrans, CA, USA). Mobile phase was the same as the previous experiments. Analysis was carried out at a flow rate of 0.27 mL/min in linear gradient of Solvent B (5–50% for 10 min; 50–75% for 3 min; 75–90% for 1 min; 90% for 3 min; 90–5% for 3 min, after that the column was re-equilibrated with 5%B for 4 min). Purity was estimated using the ChemStation for LC systems software (Agilent Technologies, Santa Clara, CA, USA).

#### 4.2.3. Peptide Concentration Measurements

Absorption spectra were recorded on a U-3210 spectrophotometer (Hitachi, Marunouchi, Chiyoda-ku, Tokyo, Japan). Aatbio tool (https://www.aatbio.com/tools/calculate-protein-concentration (accessed on 29 December 2020), AAT Bioquest, Sunnyvale, CA, USA) was used to calculate the molar extinction coefficient of NsWs at 280 nm and to calculate peptide concentrations. The total yield of peptides per 1 g of *N. sativa* seeds was calculated.

#### 4.2.4. LC/ESI-MS Analysis

Re-chromatography and mass-spectrometric analysis of the components collected after reversed-phase HPLC was performed using a LC-MS/MS API 2000 chromatograph (Applied Biosystems, Carlsbad, CA, USA). The separation was carried out using a Waters C_18_ 3 × 100 mm^2^, 3 μm (Waters, Wexford, Ireland) analytical column in isocratic condition, at a flow rate of 0.25 mL/min and monitoring of absorption at 210 nm. Sixty percent acetonitrile in 0.1% TFA was used as a mobile phase. The 50 μL of the sample was applied using an autosampler instrument. Data were obtained in a positive auto MS mode at a temperature of 250 °C and an ion spray voltage of 4500 V.

#### 4.2.5. Reduction and Alkylation

To reduce disulfide bonds, peptides were dissolved in 40 µL of 0.5 M Tris–HCl buffer containing 6 M guanidine hydrochloride and 2 mM EDTA (disodium salt), pH 8.2, and incubated at 40 °C overnight. Five microliters of 2-propanol and 4 µL of 0.7 M 1.4-dithiothreitol in water were added to the reaction mixture, vortexed and incubated at 40 °C for 4 h. For alkylation, 4 µL of 50% (*v*/*v*) 4-vinylpyridine in 2-propanol was added to the reaction mixture and incubated 20 min in the dark at room temperature. The reaction mixture was diluted with 40 mL of 0.1% TFA, and the products of the reaction were separated by RP-HPLC on a Luna C18 column (4.6 × 150 mm^2^, Phenomenex, Los Angeles, CA, USA) in a linear gradient (10–50%) of solvent B for 40 min at a flow rate of 0.75 mL/min. Peptides were detected at 214 nm. To determine the presence of free thiol groups, peptides were alkylated without preliminary reduction [23]. Each reaction contained nearly 3 nmoles of the selected peptides.

#### 4.2.6. Edman Sequencing

N-terminal amino acid sequencing was performed on an automated sequencer (PPSQ-33A model, Shimadzu Corporation, Kyoto, Japan) according to the manufacture’s protocol. Approximately 700–800 pmoles of each peptide was taken for analysis. Identification of amino acid residues (PTH-derivatives) was performed using LabSolutions software (Shimadzu Corporation, Kyoto, Japan).

### 4.3. Biological Activity

#### 4.3.1. Antifungal Activity

The spectrum of antifungal activity of the peptides and time-dependent decrease in those activity assays was evaluated in vitro by disc-diffusion assay as indicated [25,48]. All thionins tested were dissolved in 50% ethanol (*v*/*v*) and applied on discs at a concentration of 0.5 mM (~1 nmol per disc). Inhibition zones were measured manually using digital caliper. Assays were performed three times in triplicate.

#### 4.3.2. MIC Determination

Minimum inhibitory concentrations (MICs) were detected in a serial dilution assay with the purified compounds according to the European Committee on Antimicrobial Susceptibility Testing (EUCAST) protocol [49]. Tests were carried out by taking a 100 mL stock solution of the peptide in a two-fold serial dilution at concentrations ranging from 0.5 to 64 mg/mL (0.1–12 µM) in DMSO (Merck, Darmstadt, Germany). The assays were conducted in 96-well microtiter plates (BioCell Technology, Irvine, CA, USA) in RPMI 1640 (PanEco, Moscow, Russia) medium without the addition of Na_2_CO_3_. Amphotericin B (AmB) was used as a positive control; the nutrient medium was used as a negative control. Each experiment was carried out in triplicate. MIC values were defined as the lowest concentration of compounds at which the microorganisms treated demonstrated no visible growth after 48 h of incubation. Assays were performed three times in triplicate

#### 4.3.3. Cytotoxic Activity

The cytotoxicity of Nigellothionin NsW2 was determined by the MTT-test (formazan conversion assay). The following cell lines were used for the experiments: HCT-116 (human colon carcinoma, ATCC) and B16 (mouse melanoma). Human postnatal fibroblasts were used as a normal cell line. Doxorubicin was used as a positive control. Cells were cultivated in Dulbecco modified Eagle’s medium supplemented with 5% fetal calf serum, 2mM L-glutamine, 100 U/mL penicillin, and 100 mg/mL streptomycin at 37 °C in a 5% CO_2_ in a humidified atmosphere. Experiments were carried out on cells in the logarithmic phase of growth. The peptide was dissolved in DMSO as a 10 mM stock solution followed by serial dilutions in water immediately before the experiment.

Briefly, cells (5 × 10^3^ in 190 mL of culture medium) were plated in a 96-well plate and treated with 0.1% DMSO (blank control) or with nigellothionin NsW2 (0,1 to 50 µM; each concentration in duplicate) for 72 h. After incubation with the peptide, 20 µL of aqueous MTT solution (3-(4,5-dimethylthiazol-2-yl)-2,5-diphenyltetrazolium bromide, 5 mg/mL) was added into each well for 2–3 h. Formazan was dissolved in DMSO, and the absorbance at 570 nm was measured. The cytotoxicity at given NsW2 concentrations was calculated as the percentage of absorbance in wells with peptide-treated cells to that of blank control cells (100%). The IC_50_ (50% growth inhibitory concentration) was defined as the concentration of NsW2 that inhibited MTT conversion by 50%. All experiments were repeated three times, and each time in triplicate [48].

#### 4.3.4. Testing on Heterochromatin Condensation in Human Buccal Epithelium Cells

The analysis of heterochromatin granulation located in nuclei of human buccal epithelium cells was performed as described by Shckorbatov et al. [50]. The increase in heterochromatin granule quantity (HGQ) indicates the decrease in transcriptional activity in the nuclei of cells. The cells treated with 0.08–1.9 µM of NsW2, as well as control cells, were stained with orsein for 20 min. The cell nuclei were inspected by means of microscope with 400-fold magnification.

## 5. Statistical Analysis

All assays were performed in triplicate. Results were expressed as mean ± SD; *p* < 0.05 was considered statistically significant. All statistical analyses were performed using STATISTICA 6.0 (StatSoft Inc., Tulsa, OK, USA).

## 6. Conclusions

In conclusion, black cumin (*N. sativa*) seeds contain homologous antimicrobial peptides (NsW1–9). These peptides belong to the thionins family from class I and can be classified as members of a subfamily called nigellothionins based on their high similarity. A complete amino sequence was determined for three of the nigellothionins (NsW1, NsW2, and NsW4). These have low similarity with other plant thionins containing 8-cysteine motifs inside two β-turn-linked α-helices. NsW1, NsW2, and NsW4 showed significant inhibition of the collection *Aspergillus* species comparable to commercial antibiotic Amphotericin B. NsW2 was active against azole-resistant clinical isolates of *Aspergillus* and *Candida* at a micromolar range of active concentrations. We report different levels of susceptibility of the microorganisms tested to the thionin action involving a stable time-dependent decrease in activity. These effects are explained by membrane-associated interactions with the target cells. The nigellothionin NsW2 demonstrated potent cytotoxic activity against tumor and normal cell lines as well as heterochromatin condensation at active concentrations less than 1 μM. Our data provide new insights into the possible alternative mode of action different from direct interactions with the cell membrane, which might occur via cell penetration.

## Figures and Tables

**Table 1:** Initial structural analysis of the individual fractions collected after analytical reversed-phase HPLC.

Fraction	Peptide Name	Retention Time min	Molecular Mass (Average) Da	N-Terminal Amino Acid Sequence
1	NsW5	25.2	5161.6	KSCCKNTLGRNCYNTCRFMK…
2	NsW6	27.3	5161.6	KSCCKNTLGRNCYNTCRFMK…
3	NsW1	25.2	5063.4	KSCCKNTLGRNCYNTCRFMK…
4	NsW4	26.1	5041.2	KSCCKNTLGRNCYNTCRFIK…
5	NsW3	26.7	5227.3	KSCCKNTLGRNCYNTCRFIK…
6	NsW2	27.3	5141.5	KSCCKNTLGRNCYNTCRFIK…
7	NsW7	28.3	5308.4	KSCCKNTLGRNCYNTCRFMK…
8	NsW8	29.4	5271.2	GSCCKNTLGRNCYNTCRFMK…
9	NsW9	30.7	5169.6	GSCCKNTLGRNCYNTCRFIK…

**Table 2:** The activity of purified *N. sativa* thionins on *Aspergillus* strain growth (mm) measured by disc-diffusion assay. Peptide concentration is 0.5 mM (~1 nmol per disc).

Fungal Strains	Growth Inhibition Zone, mm			
	NsW1	NsW2	NsW4	Amphotericin B
*A. flavus* 3K	12.2 ± 0.4	11.0 ± 0.3	13.1 ± 0.3	13.49 ± 1.0
*A. terreus* 3K	0	0	0	0
*A. fumigatus* 2K	7.5 ± 0.1	11.1± 0.7	10.2 ± 0.6	16.8 ± 0.7
*A. oryzae* 1K	8.5 ± 0.7	12.7 ± 0.1	11.9 ± 0.8	17.5 ± 0.9

**Table 3:** Antifungal activity of the NsW2 nigellothionin measured by disc-diffusion assay (mm). * Minimum inhibitory concentration (MIC) = 0.77 µM.

Fungi	Growth Inhibition Zone, mm			
	NsW2	Amphotericin B	Nystatin	Fluconazole
Non-clinical strains				
*A. flavus* 3K	11.0 ± 0.3	13.5± 1.0	-	-
*A. fumigatus* 2K *	11.1± 0.7	16.8 ± 0.7	-	-
*A. oryzae* 1K	12.7 ± 0.1	17.5 ± 0.9	-	-
*A. terreus* 3K	0	0	-	-
Clinical isolates				
*A. fumigatus* 163m *	11 ± 0.3	-	22 ± 0.5	0
*A. flavus 905n*	11.5 ± 0.5	-	23 ± 0.3	0
*A. terreus* 1133	8.5 ± 0.3	-	15 ± 0.7	0

**Table 4:** The activity of purified NsW2 on the growth of clinical yeast strains.

Yeast Strain	Growth Inhibition Zone, mm	
	NsW2	AmB
*S. cerevisiae* 77S	12.5 ± 1	18 ± 0.3
*Cr. laurentii* 801M	11.5 ± 0.1	10 ± 0.6
*C. krusei* 1447N	7.6 ± 0.3	8.2 ± 0.8
*C. glabrata* 1402m	0	8 ± 0.3

**Table 5:** IC_50_ values of NsW2 compared with a control antitumor antibiotic (doxorubicin).

Compound	IC_50_, µM		
	HCT-116	B16	HPF
NsW2	0.30 ± 0.04	0.20 ± 0.03	0.55 ± 0.07
Doxorubicin	0.15 ± 0.02	1.20 ± 0.17	0.45 ± 0.06

## Supplementary Materials

The following are available online at https://www.mdpi.com/2079-6382/10/2/166/s1 Figure S1: Mass spectra of three major thionins from *Nigella sativa* seeds: NsW1 (A); NsW2 (B); NsW4 (C). Figure S2: Purity control of NsW1, NsW2 and NsW4 (Cromatographic profiles after analytical RP-HPLC on Phenomenex Luna C18 100×2; 3 µm; 100 Å). Figure S3: Phylogenetic tree of plant thionins based on amino acid alignment. Figure S4: Heterochromatin condensation in human buccal epithelial cells after treatment with NsW2.

## References

[1] Campos M.L., De Souza C.M., De Oliveira K.B.S., Dias S.C., Franco O.L. (2018). The role of antimicrobial peptides in plant immunity. J. Exp. Bot..

[2] Tam J.P., Wang S., Wong K.H., Tan W.L. (2015). Antimicrobial peptides from plants. Pharmaceuticals.

[3] García-Olmedo F., Molina A., Alamillo J.M., Rodriguez-Palenzuéla P. (1998). Plant defense peptides. Biopolymers.

[4] Okada T., Yoshizumi H., Terashima Y. (1970). A Lethal Toxic Substance for Brewing Yeast in Wheat and Barley: Part I. Assay of Toxicity on Various Grains, and Sensitivity Of Various Yeast Strains. Agric. Biol. Chem..

[5] Stec B. (2006). Plant thionins—The structural perspective. Cell. Mol. Life Sci..

[6] Clore G.M., Nilges M., Sukumaran D.K., Brünger A.T., Karplus M., Gronenborn A.M. (1986). The three-dimensional structure of α1-purothionin in solution: Combined use of nuclear magnetic resonance, distance geometry and restrained molecular dynamics. EMBO J..

[7] Teeter M.M., Mazer J.A., L’ltalien J.J. (1981). Primary Structure of the Hydrophobic Plant Protein Crambin. Biochemistry.

[8] Vila-Perelló M., Sánchez-Vallet A., García-Olmedo F., Molina A., Andreu D. (2003). Synthetic and structural studies on Pyrularia pubera thionin: A single-residue mutation enhances activity against Gram-negative bacteria. FEBS Lett..

[9] Llanos P., Henriquez M., Minic J., Elmorjani K., Marion D., Riquelme G., Molgo J., Benoit E. (2006). Puroindoline-a and alpha1-purothionin form ion channels in giant liposomes but exert different toxic actions on murine cells. FEBS J..

[10] Johansson S., Gullbo J., Lindholm P., Ek B., Thunberg E., Samuelsson G., Larsson R., Bohlin L., Claeson P. (2003). Small, novel proteins from the mistletoe Phoradendron tomentosum exhibit highly selective cytotoxicity to human breast cancer cells. Cell. Mol. Life Sci..

[11] Vasilchenko A.S., Smirnov A.N., Zavriev S.K., Grishin E.V., Vasilchenko A.V., Rogozhin E.A. (2017). Novel Thionins from Black Seed (*Nigella sativa* L.) Demonstrate Antimicrobial Activity. Int. J. Pept. Res. Ther..

[12] Ali S., Ganai B.A., Kamili A.N., Bhat A.A., Mir Z.A., Bhat J.A., Tyagi A., Islam S.T., Mushtaq M., Yadav P. (2018). Pathogenesis-related proteins and peptides as promising tools for engineering plants with multiple stress tolerance. Microbiol. Res..

[13] Odintsova T.I., Slezina M.P., Istomina E.A. (2018). Plant thionins: Structure, biological functions and potential use in biotechnology. Vavilovskii Zhurnal Genet. Selektsii.

[14] Bunge S., Wolters J., Apel K. (1992). A comparison of leaf thionin sequences of barley cultivars and wild barley species. MGG Mol. Gen. Genet..

[15] Schrader-Fischer G., Apel K. (1993). cDNA-derived identification of novel thionin precursors in Viscum album that contain highly divergent thionin domains but conserved signal and acidic polypeptide domains. Plant Mol. Biol..

[16] Ponz F., Paz-Ares J., Hernández-Lucas C., García-Olmedo F., Carbonero P. (1986). Cloning and nucleotide sequence of a cDNA encoding the precursor of the barley toxin α-hordothionin. Eur. J. Biochem..

[17] Castagnaro A., Maraña C., Carbonero P., García-Olmedo F. (1994). cDNA cloning and nucleotide sequences of alpha 1 and alpha 2 thionins from hexaploid wheat endosperm. Plant Physiol..

[18] Milbradt A.G., Kerek F., Moroder L., Renner C. (2003). Structural characterization of hellethionins from Helleborus purpurascens. Biochemistry.

[19] Stec B., Rao U., Teeter M.M. (1995). Refinement of purothionins reveals solute particles important for lattice formation and toxicity. Part 2: Structure of beta-purothionin at 1.7 Angstrom resolution. Acta Crystallogr. Sect. D Biol. Crystallogr..

[20] Vahedi Shahandashti R., Lass-Flörl C. (2019). Antifungal resistance in Aspergillus terreus: A current scenario. Fungal Genet. Biol..

[21] Oard S.V. (2011). Deciphering a mechanism of membrane permeabilization by α-hordothionin peptide. Biochim. Biophys. Acta Biomembr..

[22] Çakir B., Gülseren İ. (2019). Identification of Novel Proteins from Black Cumin Seed Meals Based on 2D Gel Electrophoresis and MALDI-TOF/TOF-MS Analysis. Plant Foods Hum. Nutr..

[23] Rogozhin E.A., Oshchepkova Y.I., Odintsova T.I., Khadeeva N.V., Veshkurova O.N., Egorov T.A., Grishin E.V., Salikhov S.I. (2011). Novel antifungal defensins from *Nigella sativa* L. seeds. Plant Physiol. Biochem..

[24] Oshchepkova Y.I., Veshkurova O.N., Rogozhin E.A., Musolyamov A.K., Smirnov A.N., Odintsova T.I., Egorov T.A., Grishin E.V., Salikhov S.I. (2009). Isolation of the lipid-transporting protein Ns-LTP1 from seeds of the garden fennel flower (*Nigella sativa*). Russ. J. Bioorganic Chem..

[25] Rogozhin E.A., Sadykova V.S., Baranova A.A., Vasilchenko A.S., Lushpa V.A., Mineev K.S., Georgieva M.L., Kul’ko A.B., Krasheninnikov M.E., Lyundup A.V. (2018). A novel lipopeptaibol emericellipsin a with antimicrobial and antitumor activity produced by the extremophilic fungus emericellopsis alkalina. Molecules.

[26] Orril S., Scalonis A., Urechs K., Pucci P., Schaller G. (1997). Amino Acid Sequence, SS Bridge Arrangement and Distribution in Plant Amino Acid Sequence, S-S Bridge Arrangement and Distribution in Plant Tissues of Thionins from Viscum album *. Biol. Chem..

[27] Kishi-Kaboshi M., Okada K., Kurimoto L., Murakami S., Umezawa T., Shibuya N., Yamane H., Miyao A., Takatsuji H., Takahashi A. (2010). A rice fungal MAMP-responsive MAPK cascade regulates metabolic flow to antimicrobial metabolite synthesis. Plant J..

[28] Offor B.C., Dubery I.A., Piater L.A. (2020). Prospects of gene knockouts in the functional study of mamp-triggered immunity: A review. Int. J. Mol. Sci..

[29] Slavokhotova A.A., Rogozhin E.A. (2020). Defense Peptides From the α-Hairpinin Family Are Components of Plant Innate Immunity. Front. Plant Sci..

[30] Sousa D.A., Porto W.F., Silva M.Z., Da Silva T.R., Franco O.L. (2016). Influence of cysteine and tryptophan substitution on DNA-binding activity on maize α-hairpinin antimicrobial peptide. Molecules.

[31] Nam J., Yun H., Rajasekaran G., Kumar S.D., Kim J.I., Min H.J., Shin S.Y., Lee C.W. (2018). Structural and Functional Assessment of mBjAMP1, an Antimicrobial Peptide from Branchiostoma japonicum, Revealed a Novel α-Hairpinin-like Scaffold with Membrane Permeable and DNA Binding Activity. J. Med. Chem..

[32] Rogozhin E.A., Ryazantsev D.Y., Grishin E.V., Egorov T.A., Zavriev S.K. (2012). Defense peptides from barnyard grass (*Echinochloa crusgalli* L.) seeds. Peptides.

[33] Cui X., Du J., Li J., Wang Z. (2018). Inhibitory Site of α-Hairpinin Peptide From Tartary Buckwheat Has No Effect on Its Antimicrobial Activities. Acta Biochim. Biophys. Sin. (Shanghai).

[34] Molina A., Goy P.A., Fraile A., Sánchez-Monge R., García-Olmedo F. (1993). Inhibition of bacterial and fungal plant pathogens by thionins of types I and II. Plant Sci..

[35] Bohlmann H., Clausen S., Behnke S., Giese H., Hiller C., Reimann-Philipp U., Schrader G., Barkholt V., Apel K. (1988). Leaf-specific thionins of barley-a novel class of cell wall proteins toxic to plant-pathogenic fungi and possibly involved in the defence mechanism of plants. EMBO J..

[36] Giudici M., Antonio Poveda J., Molina M.L., De La Canal L., González-Ros J.M., Pfüller K., Pfüller U., Villalaín J. (2006). Antifungal effects and mechanism of action of viscotoxin A3. FEBS J..

[37] Büssing A., Stein G.M., Wagner M., Wagner B., Schaller G., Pfüller U., Schietzel M. (1999). Accidental cell death and generation of reactive oxygen intermediates in human lymphocytes induced by thionins from *Viscum album* L. Eur. J. Biochem..

[38] Thevissen K., Terras F.R.G., Broekaert W.F. (1999). Permeabilization of fungal membranes by plant defensins inhibits fungal growth. Appl. Environ. Microbiol..

[39] Parisi K., Shafee T.M.A., Quimbar P., van der Weerden N.L., Bleackley M.R., Anderson M.A. (2019). The evolution, function and mechanisms of action for plant defensins. Semin. Cell Dev. Biol..

[40] Evett G.E., Donaldson D.M., Vernon L.P. (1986). Biological properties of Pyrularia thionin prepared from nuts of Pyrularia pubera. Toxicon.

[41] Carrasco L., Vázqes D., Hernández-Lucas C., Carbonero P., García-Olmedo F. (1981). Thionins: Plant Peptides that Modify Membrane Permeability in Cultured Mammalian Cells. Eur. J. Biochem..

[42] Hughes P., Dennis E., Whitecross M., Llewellyn D., Gage P. (2000). The cytotoxic plant protein, β-purothionin, forms ion channels in lipid membranes. J. Biol. Chem..

[43] Kul’Ko A.B., Kisil O.V., Sadykova V.S., Mikhailov V.F., Vasilieva I.M., Shulenina L.V., Zasukhina G.D., Rogozhin E.A. (2016). Investigation of thionins from black cumin *(Nigella sativa* L.) seeds showing cytotoxic, regulatory and antifungal activity. Antibiot. Khimioterapiya.

[44] Li S., Gullbo J., Lindholm P., Larsson R., Thunberg E., Samuelsson G., Bohlin L. (2002). DNA-binding domain from the mistletoe Phoradendron liga. Biochem. J..

[45] Woynarowski J.M., Konopa J. (1980). Interaction between dna and viscotoxins cytotoxic basic polypeptides from *Viscum album* L.. Hoppe Seylers Z. Physiol. Chem..

[46] OITA S. (2000). Degradation of the antimicrobial peptide alpha-thionin by Aspergillus proteases. J. Brew. Soc. JAPAN.

[47] Vasilchenko A.S., Yuryev M., Ryazantsev D.Y., Zavriev S.K., Feofanov A.V., Grishin E.V., Rogozhin E.A. (2016). Studying of cellular interaction of hairpin-like peptide EcAMP1 from barnyard grass (*Echinochloa crusgalli* L.) seeds with plant pathogenic fungus Fusarium solani using microscopy techniques. Scanning.

[48] Alferova V.A., Novikov R.A., Bychkova O.P., Rogozhin E.A., Shuvalov M.V., Prokhorenko I.A., Sadykova V.S., Kulko A.B., Dezhenkova L.G., Stepashkina E.A. (2018). Astolides A and B, antifungal and cytotoxic naphthoquinone-derived polyol macrolactones from Streptomyces hygroscopicus. Tetrahedron.

[49] Arendrup M.C., Cuenca-Estrella M., Lass-Flörl C., Hope W., Arendrup M.C., Hope W.W., Flörl C., Cuenca-Estrella M., Arikan S., Barchiesi F. (2012). EUCAST Technical Note on Aspergillus and amphotericin B, itraconazole, and posaconazole. Clin. Microbiol. Infect..

[50] Shckorbatov Y.G., Pasiuga V.N., Kolchigin N.N., Grabina V.A., Batrakov D.O., Kalashnikov V.V., Ivanchenko D.D., Bykov V.N. (2009). The influence of differently polarised microwave radiation on chromatin in human cells. Int. J. Radiat. Biol..

