# Supplementary figures and images for: Nigellothionins from Black Cumin (Nigella sativa L.) Seeds Demonstrate Strong Antifungal and Cytotoxic Activity
Source: Antibiotics (Basel). 2021 Feb 6;10(2):166. doi: 10.3390/antibiotics10020166 (PMC7914917; doi:10.3390/antibiotics10020166)

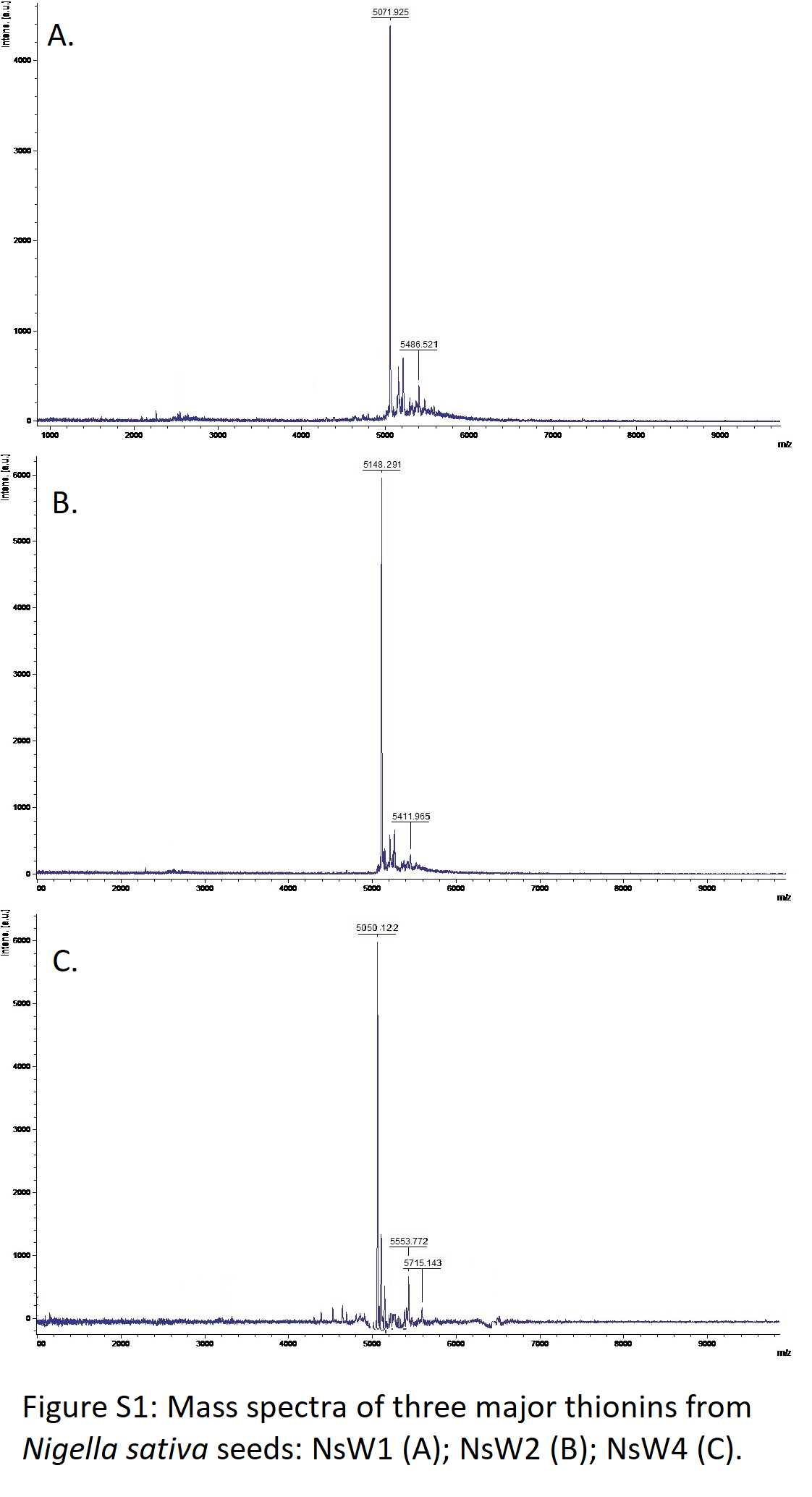

Supplement: Supplementary file 1 [file antibiotics-10-00166-s001.zip › Figure S1 Mass spectra.png]

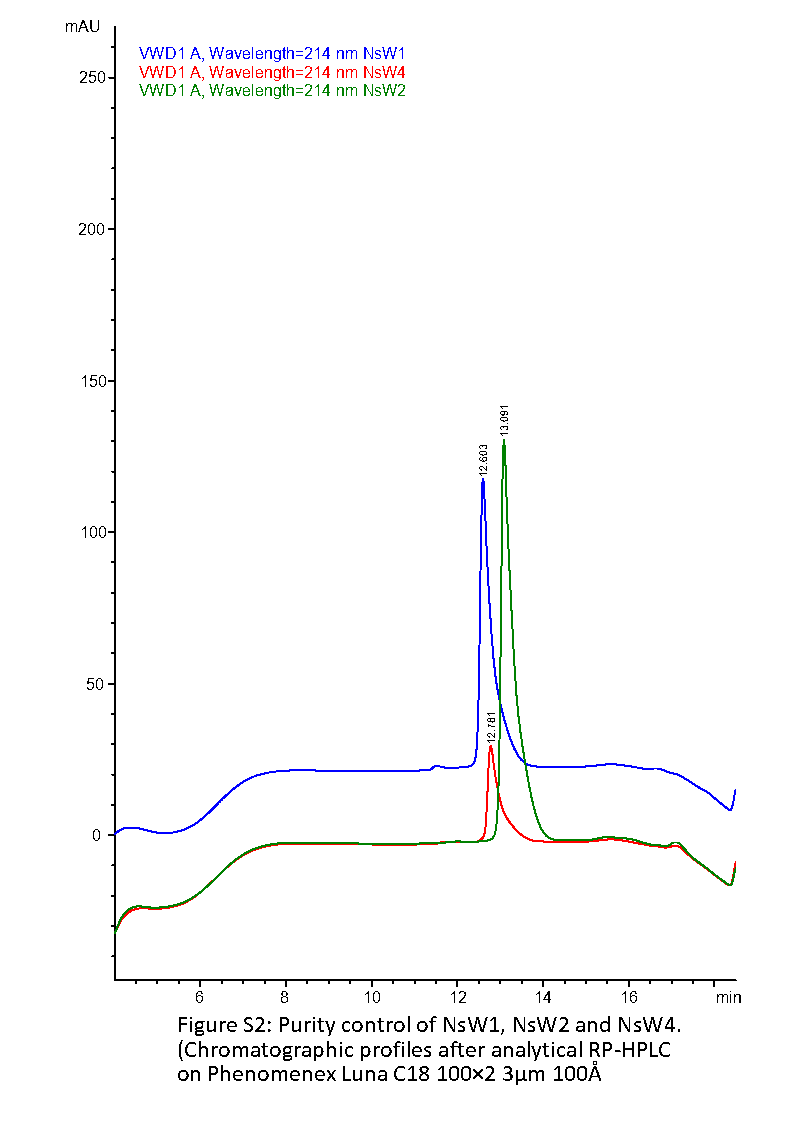

Supplement: Supplementary file 1 [file antibiotics-10-00166-s001.zip › Figure S2 Purity control.png]

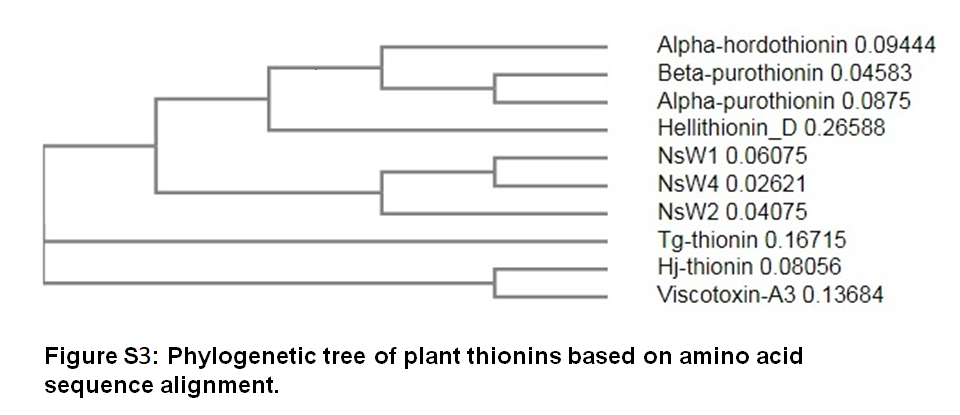

Supplement: Supplementary file 1 [file antibiotics-10-00166-s001.zip › Figure S3 Phylogenetic tree.tif]

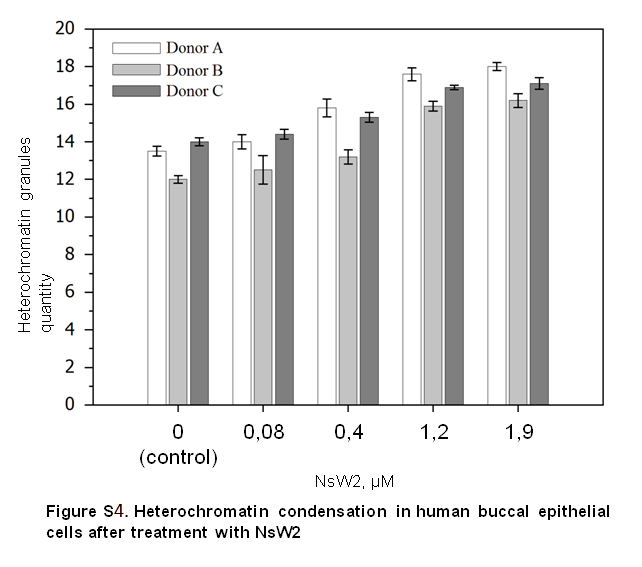

Supplement: Supplementary file 1 [file antibiotics-10-00166-s001.zip › Figure S4. Heterochromatin condensation.tif]
